# Model Corrected Blood Input Function to Compute Cerebral FDG Uptake Rates From Dynamic Total-Body PET Images of Rats *in vivo*

**DOI:** 10.3389/fmed.2021.618645

**Published:** 2021-04-07

**Authors:** James C. Massey, Vikram Seshadri, Soumen Paul, Krzysztof Mińczuk, Cesar Molinos, Jie Li, Bijoy K. Kundu

**Affiliations:** ^1^Department of Radiology and Medical Imaging, University of Virginia, Charlottesville, VA, United States; ^2^Department of Experimental Physiology and Pathophysiology, Medical University of Białystok, Białystok, Poland; ^3^Preclinical Imaging Division, Bruker Biospin, Billerica, MA, United States; ^4^Department of Biomedical Engineering, University of Virginia, Charlottesville, VA, United States; ^5^Cardiovascular Research Center, University of Virginia, Charlottesville, VA, United States

**Keywords:** arterial blood sampling, dual output model, cerebral fluoro-2-deoxy-D-glucose uptake rate, Wistar–Kyoto rat, dynamic fluoro-2-deoxy-D-glucose positron emission tomography

## Abstract

Recently, we developed a three-compartment dual-output model that incorporates spillover (SP) and partial volume (PV) corrections to simultaneously estimate the kinetic parameters and model-corrected blood input function (MCIF) from dynamic 2-[18F] fluoro-2-deoxy-D-glucose positron emission tomography (FDG PET) images of mouse heart *in vivo*. In this study, we further optimized this model and utilized the estimated MCIF to compute cerebral FDG uptake rates, *K*_*i*_, from dynamic total-body FDG PET images of control Wistar–Kyoto (WKY) rats and compared to those derived from arterial blood sampling *in vivo*. Dynamic FDG PET scans of WKY rats (*n* = 5), fasted for 6 h, were performed using the Albira Si Trimodal PET/SPECT/CT imager for 60 min. Arterial blood samples were collected for the entire imaging duration and then fitted to a seven-parameter function. The 60-min list mode PET data, corrected for attenuation, scatter, randoms, and decay, were reconstructed into 23 time bins. A 15-parameter dual-output model with SP and PV corrections was optimized with two cost functions to compute MCIF. A four-parameter compartment model was then used to compute cerebral Ki. The computed area under the curve (AUC) and *K*_*i*_ were compared to that derived from arterial blood samples. Experimental and computed AUCs were 1,893.53 ± 195.39 kBq min/cc and 1,792.65 ± 155.84 kBq min/cc, respectively (*p* = 0.76). Bland–Altman analysis of experimental vs. computed *K*_*i*_ for 35 cerebral regions in WKY rats revealed a mean difference of 0.0029 min^−1^ (~13.5%). Direct (AUC) and indirect (Ki) comparisons of model computations with arterial blood sampling were performed in WKY rats. AUC and the downstream cerebral FDG uptake rates compared well with that obtained using arterial blood samples. Experimental vs. computed cerebral *K*_*i*_ for the four super regions including cerebellum, frontal cortex, hippocampus, and striatum indicated no significant differences.

## Introduction

Noninvasive determination of blood input function from dynamic 2-[18F] fluoro-2-deoxy-D-glucose positron emission tomography (FDG PET) (Table 1) images of rodents including mice and rats has been challenging. With the availability of whole-body rodent scanners, obtaining image-derived input function (IDIF) from different sources including the inferior vena cava (IVC), left ventricular blood pool (LVBP), and the carotid arteries is possible. Due to the limited spatial resolution of the rodent scanners and the size of the regions (~1–3 mm), IDIF derived from these regions are susceptible to incomplete radioactive recovery or partial volume (PV) averaging effects and hence spillover (SP) of radioactivity into surrounding regions of interest (ROIs). Recent work by others (1) and from our laboratory has primarily utilized the LVBP (2) and the IVC (3) to derive the IDIF. The IDIF derived from the IVC is underestimated due to PV effects, while the IDIF derived from the LVBP is overestimated due to SP effects. Recent work from our laboratory has optimized a dual-output model for the myocardial tissue and the LVBP in a three-compartment kinetic model to simultaneously estimate model-corrected blood input function (MCIF) and the kinetic model parameters with SP and PV corrections to compute the rate of myocardial FDG uptake, Ki, from dynamic FDG PET images of control BL/6 mouse hearts *in vivo* (2). Recently, this model was adapted to compute myocardial *K*_*i*_ from total-body FDG PET images of control Wistar–Kyoto (WKY) and experimental spontaneously hypertensive rats (SHR) *in vivo* (4).

**Table 1:** Abbreviations.

PET	positron emission tomography
FDG	2-[18F] fluoro-2-deoxy-D-glucose
FOV	field of view
LM	list mode
MLEM	maximum-likelihood expectation maximization
IVC	inferior vena cava
LVBP	left ventricular blood pool
PV	partial volume
SP	spillover
IDIF	image-derived input function
MCIF	model-corrected input function
ROI	region of interest
Ki	rate of FDG uptake
WKY	Wistar–Kyoto
SD	Sprague–Dawley

In this study, we further optimized the dual-output model with two cost functions to compute cerebral *K*_*i*_ with MCIF derived from the LV blood pool and compared to that derived from arterial blood sampling in WKY rats *in vivo*.

## Materials and Methods

The image and blood sampling data used to support the findings of this study are available from the corresponding author upon request.

Five (*n* = 5) male WKY rats, purchased from Charles River (Kingston, NY), were housed under controlled conditions (temperature 21 ± 1°C, humidity 60 ± 10%, 12-h light/12-h dark cycle, and free access to standard rat chow and water). Animal experiments were approved by the Institutional Animal Care and Use Committee of the University of Virginia.

### Dynamic FDG PET Imaging

Five male WKY rats, 3–5 months of age, were imaged using a state-of-the-art Albira Si Trimodal PET/SPECT/CT scanner (5). The Albira PET imager is a three-ring scanner with an axial field of view (FOV) of 150 mm and trans-axial FOV of 80 mm, thereby enabling dynamic PET imaging of both the heart and the brain, in the same FOV, of 3–5-month-old rats weighing on an average of 300 g. The volumetric spatial resolution of the PET system ranges from 0.41 to 0.87 mm^3^ at the axial center with a sensitivity of 11% at the center of the FOV. The noise equivalent count rate (NECR) for the rat phantom was measured to be 240 kcps at 23 MBq (6). Dynamic FDG PET imaging of the rats, fasted for 5–6 h, was performed using a similar protocol as described recently in rats (3, 4). Briefly, a 60-min list mode (LM) acquisition, under anesthesia, was initiated, followed by intravenous injection of ~200–300 μCi of FDG via a tail vein catheter over a period of ~10 s. A three-bed CT scan followed the emission scan for attenuation correction. A small-animal gating and monitoring system (Small Animal Instruments, Inc., model 1025L for PET) was used for continuously monitoring respiration and core body temperature (using a rectal probe). The LM data were then sorted into 23 time bins [frames, time (s): 11,8; 1,12; 2,60; 1,180; 8,400], and the sinograms were reconstructed with attenuation, scatter, randoms, and decay corrections using maximum-likelihood expectation maximization (MLEM) algorithm with six iterations and 0.75-mm isotropic voxel resolution (3).

### Arterial Blood Sampling

Arterial blood sampling was performed in WKY rats following a similar protocol developed in mice (2). Twelve arterial blood samples (~0.2 ml) were collected for the entire imaging duration from the same rats more frequently at the early time points (10, 20, 36, and 49 s, 1, 1.2, 2.2, and 3.3 min) and at lower frequency at the later time points (5, 10, 30, and 60 min post FDG administration). Inactin hydrate (thiobutabarbital sodium salt hydrate) was used for our studies, as a longer duration of anesthesia was needed to perform catheterization for arterial blood sampling along with dynamic FDG PET imaging followed by CT. An incision was made under Inactin anesthesia (100 mg/kg body weight, injected intraperitoneally) in the throat area, and the carotid artery was exposed by blunt dissection. The artery was tied off above the insertion site with 4–0 silk ligature and temporarily occluded distal to the insertion site using pressure from an untied ligature. A catheter was formed from PE-50 tubing and placed in the carotid artery. The catheter was ligated with silk in three places and the pressure was removed. Blood draws were followed by flushing the catheter with a small volume of heparinized saline. Each whole-blood sample was weighed, and FDG activity was measured in a Hidex Automatic γ-Counter. The arterial blood sample data were first fitted to a seven-parameter function as described (2) to serve as the ground truth. The seven-parameter FDG blood input model has been validated by others and us for quantifying rates of FDG uptake in rodent hearts and brains. This model accurately captures the wash-in and wash-out of FDG kinetics in blood (7). The peak of this blood model was forced through the maximum tissue activity value obtained from the image-derived myocardium time activity curves (TACs) for the entire dynamic range (0–60 min) to compensate for artificially low early blood sample values as a result of limitations in blood sample timing. This was then utilized to compute experimental cerebral *K*_*i*_ using a four-parameter compartment model as described (3).

### Cerebral *K_*i*_* Modeling

Brain TACs for four super regions (cerebellum, striatum, cortex, and hippocampus) were generated through the application of the W. Schiffer T2 rat brain atlas onto dynamic PET images using PMOD (pixel-wise modeling, pmod.com). Volumes and average TACs were computed for a total of 58 volumes of interest (VOIs) consisting of 26 regions split into two different hemispheres [left (L)/right (R)] and an additional six regions without lateral split. Thirty-five of these VOIs were binned and combined to generate volumes and average TACs for the four super regions. The 58 regions generated directly from the atlas, as well as the four super regions, were analyzed in Matlab (MathWorks Inc., Natick, MA). Kinetic modeling for all regions was completed using a four-parameter compartment model (3).

### Modified Objective Function

A 15-parameter dual-output model (2) with SP and PV corrections optimized the following objective functions:

(1) $$\begin{array}{l} O_{1}(p)=\sum_{i=1}^{n}[(Model_{IDIF,i}\\ \;-PET_{IDIF,i}{)}^{2}+{\left(Model_{myo,i}-PET_{myo,i}\right)}^{2}] \end{array}$$

(2) $$\begin{array}{l} O_{2}\left(p\right)=[{\left(ModelPeak_{IDIF}-PETPeak_{IDIF}\right)}^{2}\\ \;+{\left(ModelPeak_{myo}-PETPeak_{myo}\right)}^{2}] \end{array}$$

(3) $$\begin{array}{l} O\left(p\right)=O_{1}\left(p\right)+\;O_{2}\left(p\right), \end{array}$$

where *Model*_*IDIF*_ and *Model*_*myo*_ are model output equations (Eqs. 4 and 5) and *PET*_*IDIF*_ and *PET*_*myo*_ are image-derived blood and myocardium TACs, respectively, as described (2).

(4) $$\begin{array}{l} Model_{IDIF,i}=\frac{\int_{t_{b}^{i}}^{t_{e}^{i}}\left[S_{mb}C_{T}\left(t\right)+r_{b}C_{a}\left(t\right)\right]dt}{t_{e}^{i}-t_{b}^{i}}; \end{array}$$

(5) $$\begin{array}{l} Model_{myo,i}=\frac{\int_{t_{b}^{i}}^{t_{e}^{i}}\left[r_{m}C_{T}\left(t\right)+S_{bm}C_{a}\left(t\right)\right]dt}{t_{e}^{i}-t_{b}^{i}}. \end{array}$$

*S*_*mb*_ and *S*_*bm*_ are SP contamination factors from the myocardium to the blood and vice versa; *r*_*b*_ and *r*_*m*_ are recovery coefficients (RCs) for blood pool and myocardium, respectively, and *t*_*b*_ and *t*_*e*_ are the beginning and end of a time frame. *C*_*T*_(*t*), the model tissue, was obtained by solving FDG transport differential equations from blood to tissue spaces (2). *C*_*a*_(*t*) is the seven-parameter model blood for FDG transport as described (2, 7).

The second objective function, *O*_2_(*p*), minimizes the square of the difference between the model and image-derived blood and myocardium peak values. The *ModelPeak* in Eq. (2) was computed from the model equations for the IDIF (*Model_IDIF*) and myocardium (*Model_myo*) (Eqs. 4 and 5, respectively). The *PETPeak* values were derived from the dynamic PET images for both the blood and the myocardium for each rat. Optimization of the objective function, *O*(*p*), using nonlinear regression analysis written in Matlab, resulted in the estimation of MCIF, which was then used to compute cerebral *Ki*, defined as:

(6) $$\begin{array}{l} K_{i}=\frac{K_{1}k_{3}}{\left(k_{2}+k_{3}\right)}, \end{array}$$

using a four-parameter compartment model,

(7) $$\begin{array}{l} C_{m}\left(t\right)=\frac{1}{\left(t_{2}-t_{1}\right)}\int_{t_{1}}^{t_{2}}\left\{(1-TBV\right)[\frac{K_{1}k_{3}}{k_{2}+k_{3}}\int_{0}^{T}C_{p}\left(u\right)du\\ \;+\frac{K_{1}k_{2}}{k_{2}+k_{3}}\int_{0}^{T}C_{p}\left(u\right).e^{-\left(k_{2}+k_{3}\right)\left(T-u\right)}du]+TBV.C_{p}(T)\}\;dT, \end{array}$$

as described (3).

The computed area under the curve (AUC under MCIF) and *K*_*i*_ were compared to those derived from arterial blood samples. We refer to AUC as direct and downstream *K*_*i*_ as indirect comparisons.

### Statistical Analysis

All data were reported as mean ± SE. Bland–Altman analysis was used to assess the agreement between experimental and computed cerebral *K*_*i*_ for 35 VOIs. Computed and measured AUC and downstream *K*_*i*_ of the four super regions were compared using paired Student's *t-*tests. A *p*-value of <0.05 was considered statistically significant.

## Results

**Figure 1:**
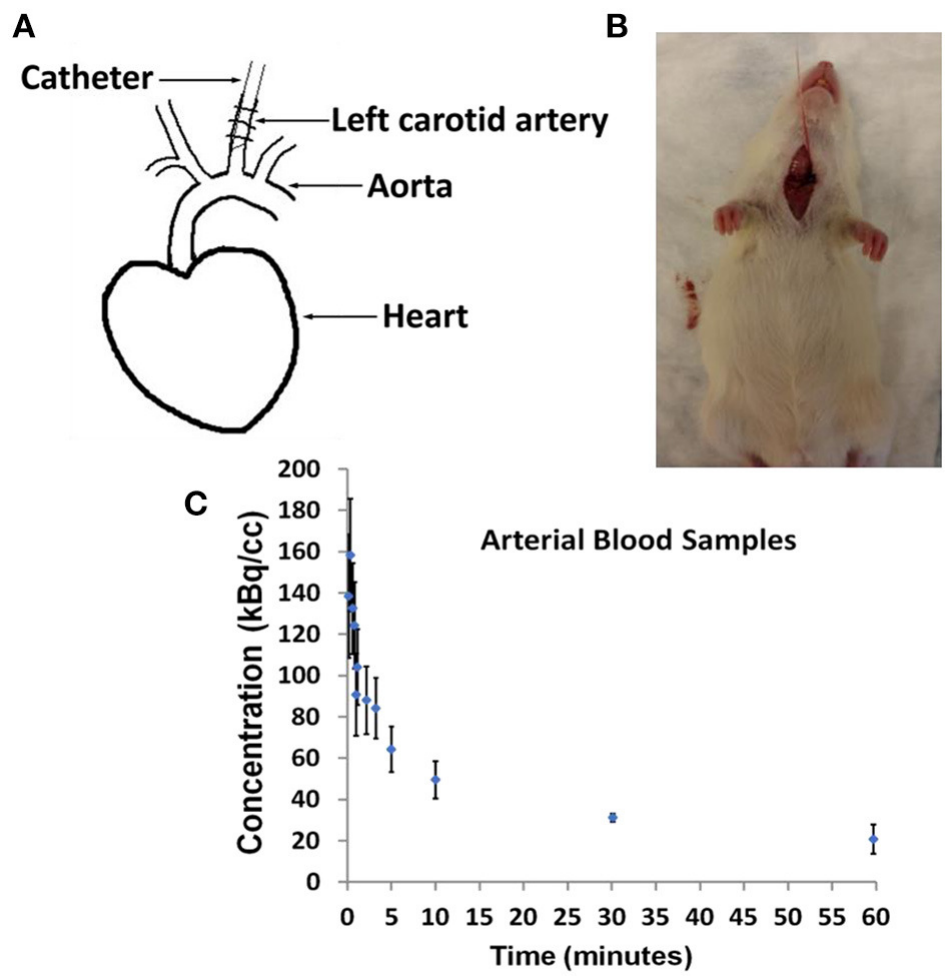
Arterial Blood Sampling in Wistar–Kyoto (WKY) rats. **(A)** Sketch showing catheterization of the carotid artery. **(B)** Picture of a WKY rat during the experimental procedure. **(C)** Arterial Blood Sample measurements averaged over *n* = 5 WKY rats with standard error as a function of image acquisition time.

In Figure 1, we show arterial blood sampling data in WKY rats. A sketch of the experimental arterial catheterization setup is shown in Figure 1A. In Figure 1B, example picture of carotid artery catheterization is shown in a WKY rat. Average activity (with standard error) normalized by the injected dose and the weight of the animal for five WKY rats are shown in Figure 1C.

**Figure 2:**
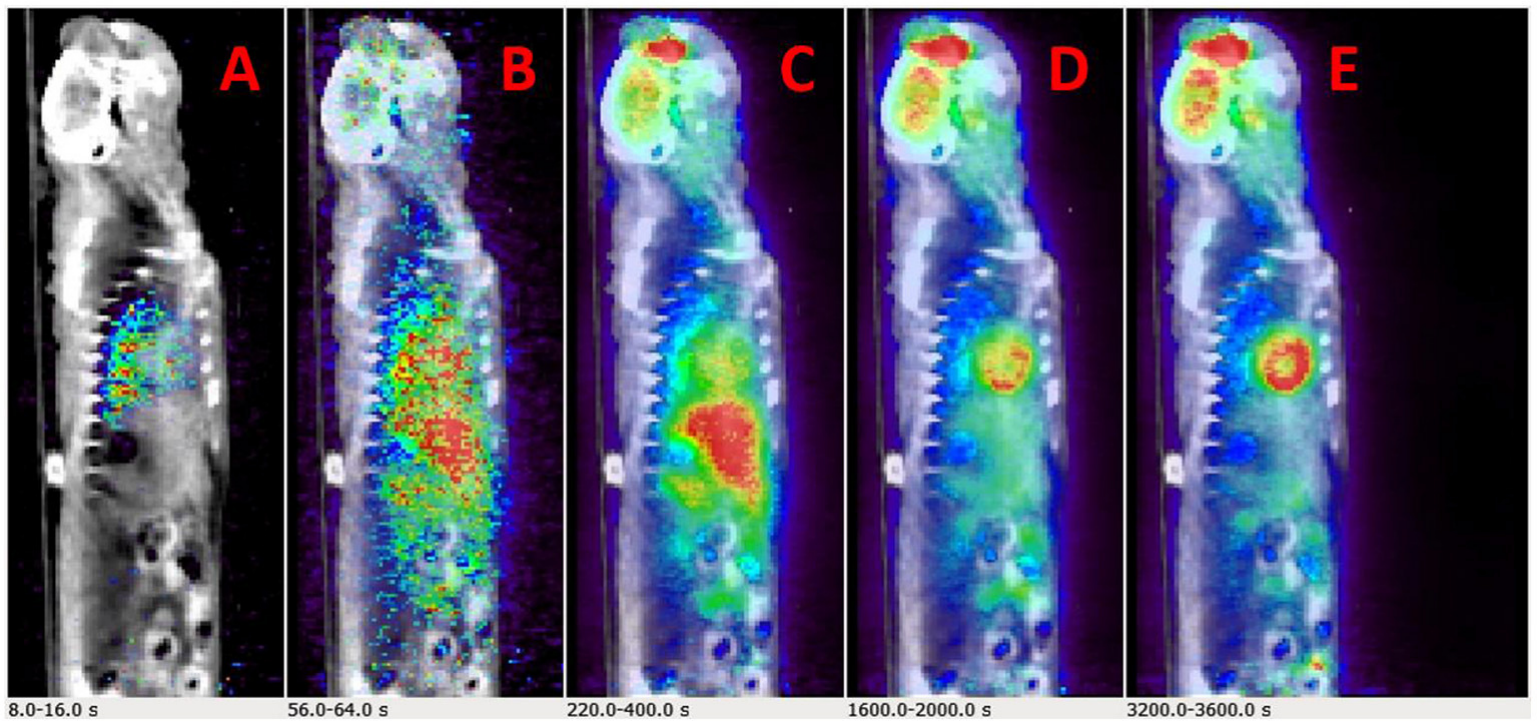
Time-resolved total-body 2-[18F] fluoro-2-deoxy-D-glucose positron emission tomography (FDG PET) images. **(A–E)** Example PET/CT rendering cut across showing uptake in Wistar–Kyoto (WKY) rat myocardium and brain over a period of 60 min.

Example dynamic FDG PET images co-registered with CT over a period of 60 min are shown in Figures 2A–E. The images show that FDG traverses from the vascular space at the early time points (Figures 2A,B) at ~1 min, to the extravascular spaces (Figure 2C) at ~5 min, and gets trapped as FDG-6-phosphatase in the tissue space including the heart and the brain at the late time points (Figures 2D,E) between 30 and 56 min post FDG administration.

**Figure 3:**
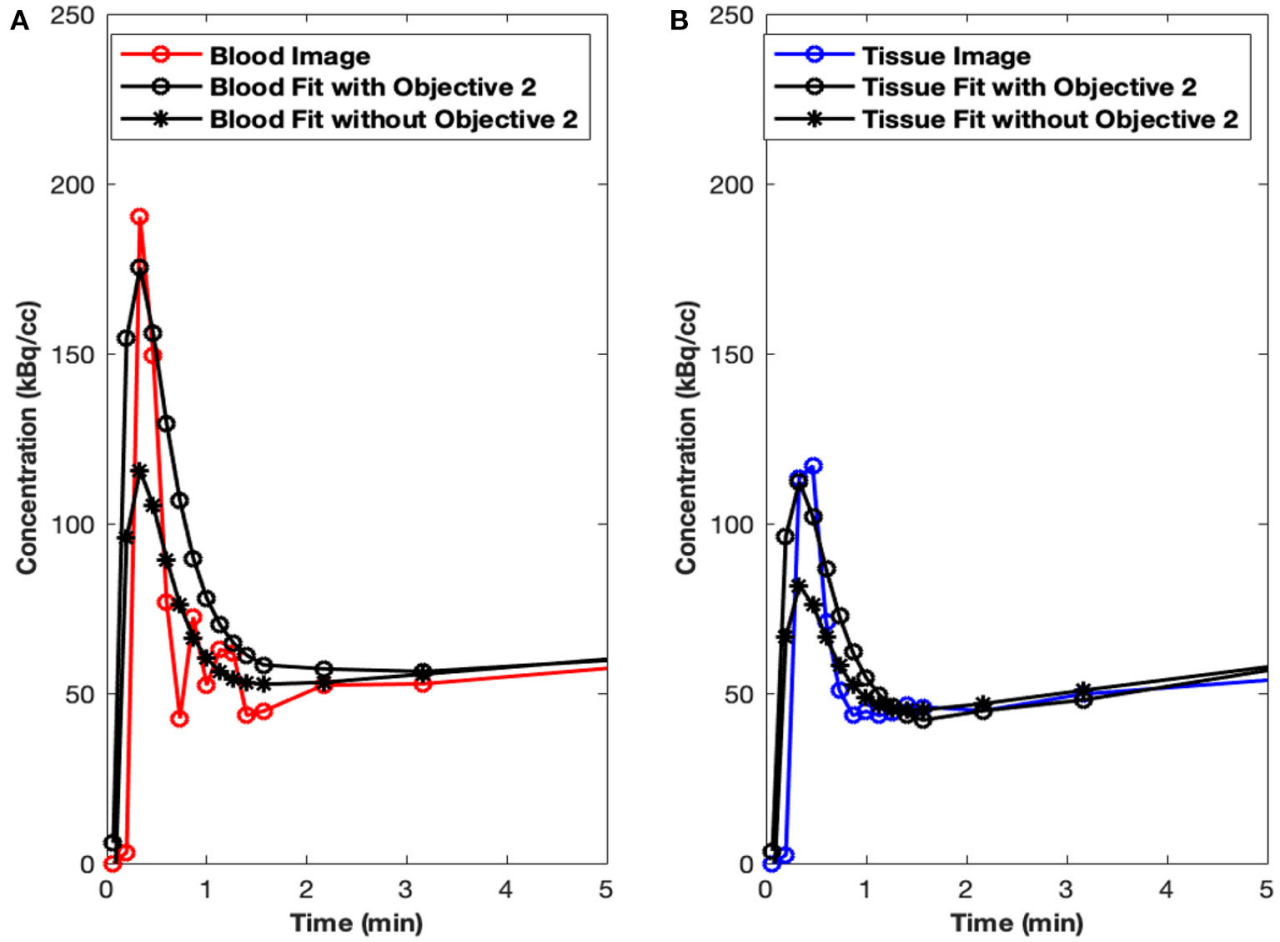
Peak fits with objective function O_2_(p). **(A)** Peak blood fit with and without the second objective function, O_2_(p) Equation (2). **(B)** Peak tissue fit with and without O_2_(p).

Initial runs of the optimization using just the first objective term, *O*_1_(*p*) (Eq. 1), showed fits with relatively poor matching of the peak values at the early time points for both tissue (myocardium) and blood data. We hypothesized that this may have been due to the fact that the points are all weighted equally under *O*_1_(*p*), which meant that the matching of peak values may have been sacrificed by the algorithm in favor of later time point values, which are more numerous. Knowing that first-pass behavior is a crucial component of dynamic tracer analysis, we introduced a second objective function, *O*_2_(*p*) (Eq. 2), to create models that more accurately reflect the behavior of this key system component. By introducing *O*_2_(*p*), we weigh the early time points more heavily than others, which gives more equal distribution of emphasis during optimization. This resulted in more accurate fitting of peak values, without significantly lowering the late time point fits as illustrated in Figure 3A (peak blood fit) and Figure 3B (peak tissue fit). Since the blood peaks at the earlier time points (decays with time), forcing the maximum of the model peak to the maximum of the image-derived peak results in more accurate fits at the same time point. However, since the myocardium TACs derived from the images have a lower peak uptake (SP contamination from the blood to the myocardium) at the early time points and higher uptake 10–15 min and beyond (trapping of FDG into the myocardium), forcing the maximum of the Model_myo to the maximum of the image-derived values within the first 10 min results in a slight mismatch in the peak fits for the myocardium.

**Figure 4:**
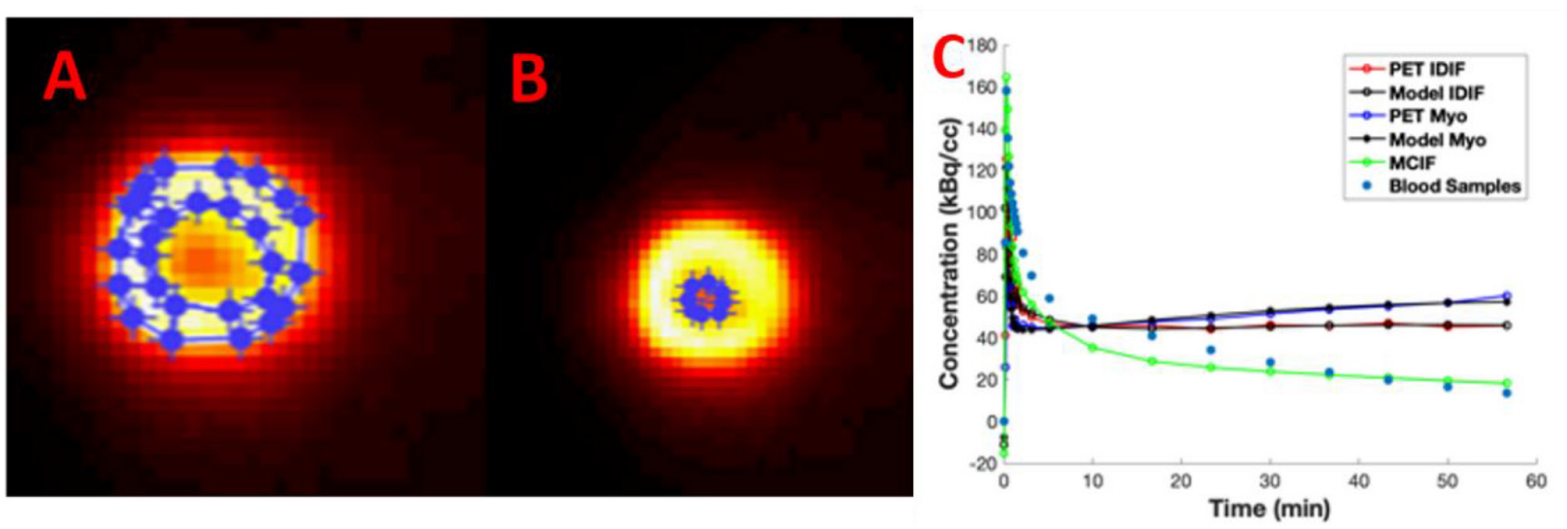
Model-corrected blood input function (MCIF). **(A)** Representative 2-[18F] fluoro-2-deoxy-D-glucose positron emission tomography (FDG PET) images with regions of interest drawn in the myocardium and **(B)** left ventricular blood in the last time frame. **(C)** Dual-output model (Model IDIF, Model Myo) fitted to image-derived blood (PET IDIF) and myocardium (PET Myo) time activity curves (TACs) to generate the MCIF. For comparison, blood samples derived from arterial blood sampling are shown.

In Figures 4A–C, we show example ROIs and model calculations to compute MCIF. Example ROIs in the myocardium and the LVBP at the last time frame are shown in Figures 4A,B. TACs for the myocardium and LV blood pool obtained from dynamic FDG PET images of the heart and model calculations in a dual-output model to compute MCIF along with arterial blood samples for the entire imaging duration of 60 min are shown in Figure 4C. The AUC (direct) for MCIF averaged over five WKY rats was computed to be 1,792.65 ± 348.48 kBq min/cc and that for the arterial blood samples was measured to be 1,893.53 ± 436.91 kBq min/cc (*p* = 0.76). Although the blood inputs were not significantly different, point by point, the MCIF was consistently lower than the experimental blood input by 6.18% on average relative to the experimental curve.

**Figure 5:**
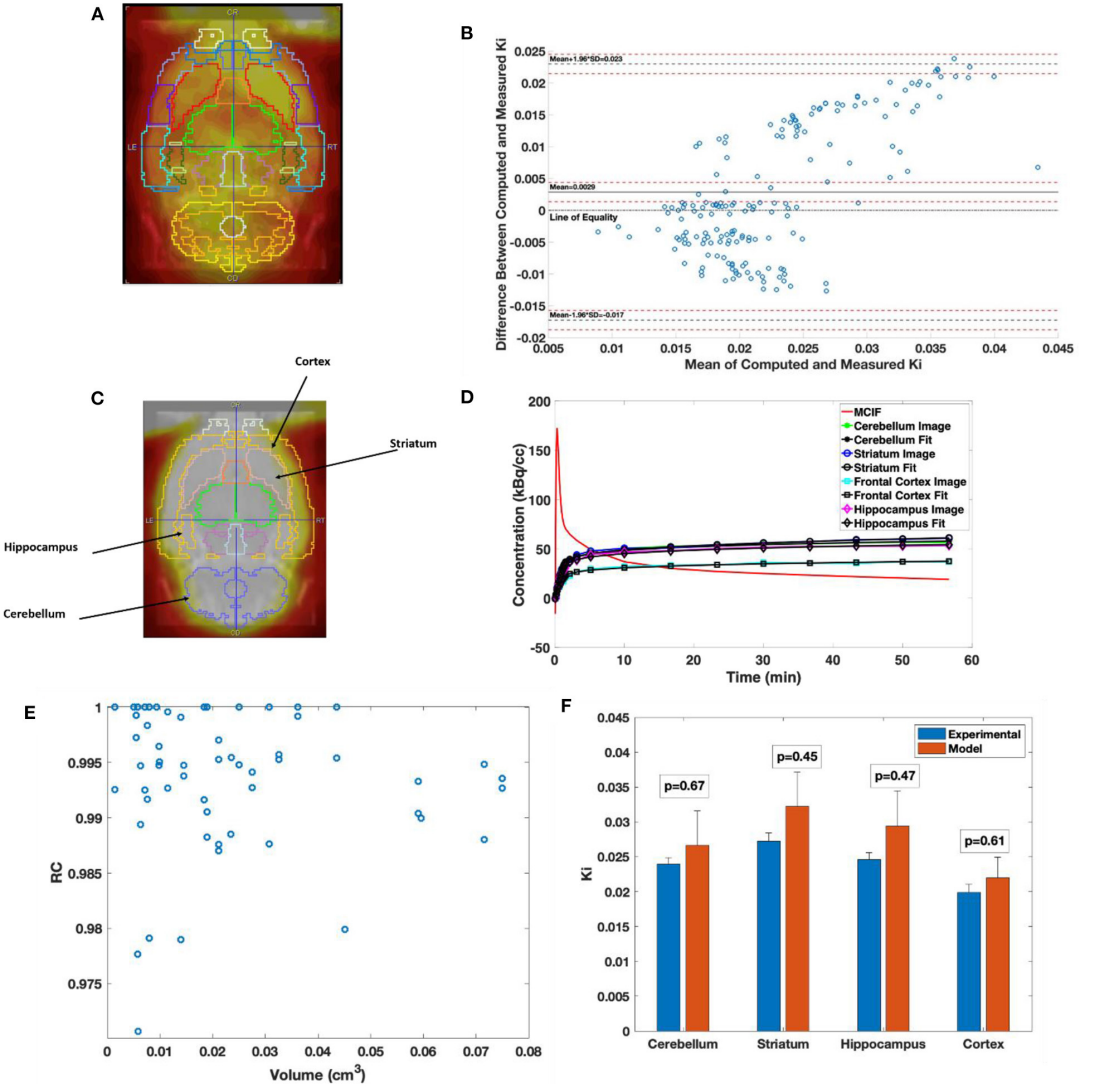
Computed cerebral 2-[18F] fluoro-2-deoxy-D-glucose (FDG) uptake rates in Wistar–Kyoto (WKY) rats. **(A)** Example brain PET image of WKY rat co-registered onto W. Schiffer T2 rat brain atlas showing 35 volumes of interest. **(B)** Bland–Altman plot comparing computed model and experimental Ki. **(C)** Example brain PET image of WKY rat co-registered onto W. Schiffer T2 rat brain atlas combining the 35 volumes of interest (VOIs) into four regions of interest. **(D)** Four-parameter compartment model fitted to time activity curves (TACs) derived from the dynamic PET data for the regions indicated in panel **C**. **(E)** Recovery coefficient (RC) of all the 58 VOIs derived from the atlas for an example WKY rat. **(F)** Comparison between experimental and computed *K*_*i*_ for the cerebellum, striatum, hippocampus, and frontal cortex.

In Figure 5A, the W. Schiffer T2 rat brain atlas fused with a coronal slice of a late time point dynamic PET image is shown for an example WKY rat. Bland–Altman analysis was used to compare the two methods, including tests for similarity and model bias. Bland–Altman analysis between computed and measured *K*_*i*_ for the 35 VOIs contributing to the four super regions for five WKY rats (175 data points) revealed a mean difference of 0.0029 min^−1^ (~13.5%). The precision (standard deviation of differences) was 0.0103 min^−1^. All differences were inside the lower and upper limits of agreement (mean ± 1.96 SD), which were −0.0172 and 0.0230, respectively (Figure 5B). The plot appeared to show a trend of increasing error with increasing magnitude of *K*_*i*_, which necessitated testing for bias to ensure there were no inherent biases beyond the level of the insignificantly underestimated blood input function. The 95% confidence interval limits for mean and two standard deviation-based agreement limits revealed that the mean difference was likely due to a systematic bias from MCIF underestimating the blood sample inputs, with the line of equality falling outside the confidence interval for the mean percentage difference; however, 98.8% of measurements remained within the limits of agreement despite this bias. The four super regions (formed by combining the above 35 VOIs), namely, cerebellum, striatum, cortex, and hippocampus are outlined for a WKY rat in Figure 5C. Example TACs obtained from the dynamic FDG PET images of the four super regions, model calculations, and MCIF are shown in Figure 5D. The RC in Eq. (5) is the term (1–TBV). In Figure 5E, we plot RC as a function of volume, where we show that the model computations result in radioactivity recovery between 0.97 and 1 for all the brain structures (58 data points). RCs for the four super regions (combining 35 of the 58 data points) were close to 1 (data not shown). Computed *K*_*i*_ and measured *K*_*i*_ for the four super regions are shown in Figure 5F. Although the underestimation of the blood input led to a small systematic bias factor, leading to slight overestimation of *K*_*i*_ by the model, no differences for any region were statistically significant. Experimental and computed *K*_*i*_ for the cerebellum were 0.024 ± 0.001 and 0.027 ± 0.01 min^−1^, respectively (*p* = 0.67); for the hippocampus were 0.025 ± 0.001 and 0.029 ± 0.01 min^−1^, respectively (*p* = 0.47); for the frontal cortex were 0.019 ± 0.001 and 0.022±0.004 min^−1^, respectively (*p* = 0.60); and for the striatum were 0.027 ± 0.001 and 0.032±0.01 min^−1^, respectively (*p* = 0.45), thereby indicating no significant differences.

## Discussion

Arterial blood sampling, although invasive in nature, is the gold standard for obtaining the blood input function to compute myocardial or cerebral FDG uptake rates in dynamic FDG PET scans of rodents (8). Several image-derived methods have been developed over the last several years for noninvasive determination of the blood input function. One such method is the hybrid method (9) that relies on image-derived sampling from the LVBP at the early time points due to the rapid change in FDG metabolism and invasive arterial or venous blood sampling at the late time points. Another method (10–12) employed factor analysis techniques using principle component analysis (PCA) to segment out LVBP from the myocardium and the surrounding background to compute image-derived blood input function based on time-dependent pixels in dynamic FDG PET scans. However, the former method overestimates and the latter method underestimates the computed myocardial *K*_*i*_ due to significant SP-out of radioactivity from the blood to the tissue at the early time points and SP-in from the tissue to the blood at the late time points, respectively. Recent work by others and from our laboratory utilized the IVC as a possible source of deriving the blood input function for computing cerebral (13) and myocardial (3) *K*_*i*_. Both these methods require either arterial blood sampling for dispersion correction (13) or structural information for PV corrections (3).

Recently, a 15-parameter dual output model was developed in a three-compartment model that simultaneously estimates the MCIF derived from the LVBP with SP and PV corrections and the kinetic parameters for determination of myocardial and cerebral *K*_*i*_ from dynamic FDG PET images of mouse and rat brain and heart *in vivo* (1, 2, 4). The optimization method here used “fmincon” in Matlab, which utilizes a deterministic algorithm based on interior-reflective Newton (IRN) algorithm to search for a local minimum. One method (1) utilizes a blood sample in the optimization of their objective function, while our methods optimize the same objective function (Eq. 1) on high-resolution ordered subset expectation maximization (OSEM) dynamic gated images of mouse hearts (2) and high-resolution MLEM dynamic images of rat hearts (3, 4), which possibly obviates the need for any blood samples in the optimization. In this study, we used this and further minimized the differences between the model and image-derived peak values (Eq. 2) to estimate MCIF without the need for any arterial blood samples as optimization parameters. This MCIF was further utilized to compute cerebral FDG *K*_*i*_ and also compared to that derived using arterial blood samples in WKY rats. The multiparameter nature of the model may introduce uncertainties in the computed results using nonlinear regression. The initial guesses and bounds for the PV averaging coefficients (Eqs. 4 and 5) were determined from the phantom experiments (RC plot) performed previously in our laboratory (2, 3). The dimensions of the myocardial wall and the left ventricle were based on our recent MRI measurements in WKY rats (4). We kept the bounds for SP factors (Eqs. 4 and 5) open between 0 and 1, as they are a function of time. As for the model blood input function, one side of the bounds was determined by the distribution of the input function and the other side was left wide open as described (2, 14).

It has been recently shown that for structures ranging between 4 and 5 mm and beyond the RC for six MLEM iterations is between 0.6 and 0.85. The RC plateaus out between 0.8 and 0.95 for similar structures at 10 iterations (15). Additionally, we perform partial volume (1-TBV) and spillover (TBV) corrections during modeling as shown in Eq. (5), resulting in radioactivity recovery between 0.97 and 1 for all the brain structures (Figure 5E). Since the brain structures in the study are >2 times the full width half maximum (FWHM) spatial resolution of the scanner, we expect 100% recovery of the radioactivity. However, due to minimal TBV (SP contamination in the tissue), the radioactivity recoveries (1-TBV) for all the brain structures are close to 100% (97% and greater). This model however assumes that the SP and PV (TBV and 1-TBV) add up to 1. A future computation will consider these as independent parameters. We also find for example WKY rat data that the downstream *K*_*i*_ with SP and PV corrections for an image reconstructed with six iterations vs. 24 iterations are very similar (data not shown), indicating stability of the computed values.

A recent review article (16) has identified WKY and Sprague–Dawley (SD) rats as commonly used strains in both cardiac PET and SPECT imaging studies. Previous studies have computed and measured cerebral *K*_*i*_ in SD rats (1, 11). Our computed and measured cerebral *K*_*i*_ in WKY rats are higher than those from SD rats in one case (1) and lower than those in the other (11), although both these studies performed their arterial blood sampling and imaging under isoflurane anesthesia under fed conditions (unfasted rats). Anesthesia plays an important role in tissue FDG uptake in a rodent PET scan especially in the myocardium (four-fold higher uptake for isoflurane vs. ketamine/xylazine). The effect of anesthesia on rodent cerebral FDG uptake, however, is only ~1.3-fold higher using isoflurane compared to that using ketamine/xylazine (17). Fasting duration also affects tissue uptake in an FDG PET scan (18). Whereas, our studies fasted the WKY rats for ~5–6 h, the other studies in SD rats were in fed state (unfasted). Additionally, one of the studies performed attenuation correction using a Co-57 transmission scan (1) compared to CT-based attenuation correction in our study, whereas the other study did not perform any attenuation correction (11). Thus, the differences in the measured cerebral *K*_*i*_ between the earlier studies and ours may possibly be attributed to a combination of the differences in the mode of attenuation correction (transmission based vs. CT based vs. no attenuation correction), anesthetic used (isoflurane vs. Inactin), fasting state (fed vs. unfed), and strain of the rats (WKY vs. SD). A limitation of this study is the sample size. Since the studies were performed in control WKY rats, we do not expect much variation in the metabolic changes in these rats. However, it should be noted that this study is a validation of the methodology for computing the rate of cerebral FDG uptake using input function from the LVBP with SP and PV corrections for the first time *in vivo*.

## Conclusions

Direct and indirect comparisons of model computations with arterial blood sampling were performed for the first time in WKY rats. MCIF computed from the LVBP compared well with arterial blood samples. Computed cerebellum, frontal cortex, hippocampus, and striatal FDG uptake rates agreed well with those obtained from arterial blood samples.

## Data Availability Statement

The original contributions presented in the study are included in the article/Supplementary Material, further inquiries can be directed to the corresponding author/s.

## Ethics Statement

The animal study was reviewed and approved by Institutional Animal Care and Use Committee University of Virginia.

## Author Contributions

JM performed modeling of image data. BK conceptualized the study, performed PET imaging, and modeling of image data. BK is the corresponding author. JL performed PET imaging and arterial blood sampling. CM performed image reconstruction. KM performed PET imaging and arterial blood sampling. SP performed PET imaging. VS performed modeling of image data. All authors contributed to the article and approved the submitted version.

## Conflict of Interest

CM was employed by company Bruker Biospin.

The remaining authors declare that the research was conducted in the absence of any commercial or financial relationships that could be construed as a potential conflict of interest.
